# Genetic Architecture of Micro-Environmental Plasticity in *Drosophila melanogaster*

**DOI:** 10.1038/srep09785

**Published:** 2015-05-06

**Authors:** Fabio Morgante, Peter Sørensen, Daniel A. Sorensen, Christian Maltecca, Trudy F. C. Mackay

**Affiliations:** 1 Department of Biological Sciences and W. M. Keck Center for Behavioral Biology; 2 Program in Genetics; 3 Department of Animal Science, North Carolina State University, Raleigh NC 27695-7614 USA; 4 Center of Quantitative Genetics and Genomics and Department of Molecular Biology and Genetics, Aarhus University, Tjele 8830, Denmark

## Abstract

Individuals of the same genotype do not have the same phenotype for quantitative traits when reared under common macro-environmental conditions, a phenomenon called micro-environmental plasticity. Genetic variation in micro-environmental plasticity is assumed in models of the evolution of phenotypic variance, and is important in applied breeding and personalized medicine. Here, we quantified genetic variation for micro-environmental plasticity for three quantitative traits in the inbred, sequenced lines of the *Drosophila melanogaster* Genetic Reference Panel. We found substantial genetic variation for micro-environmental plasticity for all traits, with broad sense heritabilities of the same magnitude or greater than those of trait means. Micro-environmental plasticity is not correlated with residual segregating variation, is trait-specific, and has genetic correlations with trait means ranging from zero to near unity. We identified several candidate genes associated with micro-environmental plasticity of startle response, including *Drosophila Hsp90*, setting the stage for future genetic dissection of this phenomenon.

Morphological, physiological, behavioral and fitness-related traits typically exhibit continuous phenotypic variation in populations, often approximating a normal distribution. In classical quantitative genetics, this variation is posited to arise from two independent sources^1^,^2^. The genetic component of variation results from the segregation of alleles at multiple loci with effects that are generally too small to be identified via their segregation in pedigrees, and can be further partitioned into additive, dominant and epistatic effects. The environmental component of variation arises because a single genotype can give rise to multiple phenotypes due to small fluctuations in the developmental, physical and social environment to which individuals with the same genotype are exposed.

However, the genetic and environmental components of phenotypic variation may not be independent. A common feature of quantitative traits is that they exhibit phenotypic plasticity, such that the mean phenotype of a population changes if there is a shift in the environmental conditions (*e.g*., temperature) to which it is exposed. The magnitude of plasticity with respect to the population mean can be readily estimated if the environmental change can be replicated (a macro-environmental perturbation) and the same constellation of genotypes is reared under all environments, and visualized as a norm of reaction^1^,^2^. Further, if such a study is repeated with multiple defined genotypes, we can assess whether there is variation in the shape of the reaction norms across genotypes. If so, there is genotypic variation in macro-environmental plasticity, or genotype by environmental interaction, which is formally equivalent to a departure in the cross-environment genetic correlation from unity^3^. The existence and evolutionary significance of genotype by environment interaction is well-documented^4^,^5^,^6^,^7^,^8^.

Phenotypic plasticity can also occur within a defined macro-environment. Micro-environmental plasticity manifests as variation among individuals of the same genotype reared in a common environment. Micro-environmental plasticity has been variously (and confusingly) called non-genetic variance, developmental noise, stochastic variation, environmental sensitivity, residual variance and general environmental variance^1^,^9^,^10^. The converse of micro-environmental plasticity is robustness, homeostasis, developmental stability or environmental canalization^1^,^9^,^10^. Another manifestation of micro-environmental plasticity is between spatially or temporally repeated traits within an individual (special environmental variance, or fluctuating asymmetry)^1^,^9^,^10^,^11^. Here, we are concerned with the former manifestation; *i.e*., between individuals.

Genetic variance in micro-environmental plasticity is assumed in evolutionary models of the evolution of phenotypic variance^12^,^13^,^14^,^15^,^16^,^17^,^18^,^19^,^20^,^21^. These models predict the evolution towards an optimum of zero environmental and genetic variance for quantitative traits under constant stabilizing selection^12^,^14^,^15^,^16^,^21^. Hypotheses for why the environmental variance never reaches the optimum (a canalization limit) include a correlation between the effects of mutations conferring robustness with direct effects on the same trait^16^; deleterious pleiotropic effects of mutations affecting robustness^16^; a fitness cost for environmental homogeneity^19^; and mutation-stabilizing selection balance if there is a bias towards new mutations increasing the environmental variance^20^. However, if a trait is under stabilizing selection and the optimum fluctuates between generations, there will be selection favoring increased micro-environmental variance^12^,^13^,^14^,^18^,^19^,^21^. This is because increased environmental variance will maximize fitness by ensuring more individuals are at or near the current optimum (a ‘bet-hedging’^14^ or ‘adaptive coin-flipping’ strategy^13^). Finally, strong directional selection favoring extreme individuals is expected to increase micro-environmental plasticity^17^,^22^.

From the applied breeding perspective, where uniformity of the final product is important, the existence of genetic variation for micro-environmental variance implies that selection for reduced micro-environmental plasticity may be successful in achieving this goal^17^,^22^,^23^,^24^. Finally, from the perspective of personalized medicine, genetic variation in micro-environmental plasticity could contribute to variable penetrance and expressivity of risk alleles.

Quantifying the magnitude of genetic variance of micro-environmental plasticity is challenging, as it involves considering the residual variance (or the residual standard deviation) as a trait and using linear mixed model methodology to disentangle genetic effects acting on it^22^. Larger sample sizes are needed to accurately estimate variance compared to a mean; the non-normal distribution of the variance necessitates transforming the data to restore normality^22^,^25^,^26^,^27^,^28^; and the within-family variance confounds micro-environmental with genetic variance in classic outbred family-based designs^22^. Nevertheless, there is growing evidence for a genetic component of micro-environmental plasticity from outbred family-based analyses for milk yield in dairy cows^29^; litter size in sheep^25^, pigs^26^,^28^, rabbits^28^ and mice^30^; and body weight in snails^31^, chickens^32^ and pigs^33^.

Inbred line designs are more powerful than family-based designs for estimating the genetic basis of micro-environmental plasticity, because all variance between individuals within a completely homozygous inbred line is due to micro-environmental plasticity. Indeed, genetic variation of micro-environmental plasticity has been documented for wing size and shape^34^, numbers of sensory bristles^35^ and sleep traits^36^ in *Drosophila melanogaster*; fitness and morphological traits in *Arabidopsis thaliana*^37^,^38^; morphological and fitness traits in maize^39^; and gene expression in *Saccharomyces cerevisiae*^40^. Although determining the genetic loci and molecular polymorphisms affecting genetic variation for micro-environmental plasticity is in its infancy, several of these studies mapped quantitative trait loci^37^,^38^,^39^,^40^ and identified candidate genes^35^,^36^,^37^,^38^,^40^ affecting micro-environmental plasticity.

Here, we used the sequenced, inbred lines of the *D. melanogaster* Genetic Reference Panel (DGRP)^41^,^42^ to quantify and evaluate the significance of genetic variation in micro-environmental plasticity for starvation resistance^42^, time to recover from a chill-induced coma^41^ and startle response^43^. We found levels of genetic variance for micro-environmental variance equivalent to those for the trait means. We then mapped common molecular variants associated with micro-environmental plasticity, implicating *Hsp90* as well as novel candidate genes.

## Results

### Highly heritable variation for micro-environmental plasticity

First, we tested whether there was heterogeneity of micro-environmental variance among lines, using both Brown-Forsythe and Levene’s tests (modifications of one-way analysis of variance (ANOVA) that use, respectively, the absolute deviation of each data point from the line median and mean as the response variable). We found highly significant heterogeneity of micro-environmental variance for all three traits in both sexes (Table 1).

Next, we assessed the suitability of three metrics and their natural logarithms for quantifying micro-environmental plasticity: the within-line standard deviation (*σ_E_*, ln(*σ_E_*)); the within-line coefficient of environmental variation (*CV*_*E*_, ln(*CV*_*E*_)) and the within-line median absolute deviation (*MAD*, ln(*MAD*)). We estimated these metrics for three quantitative traits – time to recover from a chill-induced coma, starvation stress resistance, and startle response – for 174-201 largely homozygous DGRP lines^42^. ln(*σ_E_*) and ln(*CV*_*E*_) were nearly normally distributed for all analyses; the other metrics were highly skewed (Table S1, Figures S1-S3). We used ln(*σ_E_*) as our metric of micro-environmental variation because it is perfectly correlated with ln(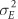), which has desirable statistical properties and has been used previously to quantify residual variance^32^,^39^; and ln(*σ_E_*) has the advantage of being on the same scale as the trait mean. Further, the product moment correlations between ln(*σ*_*E*_) and ln(*CV*_E_) are high for all traits. In females, the correlations between these metrics are 0.87, 0.81 and 0.70 for chill coma recovery time, startle response, and starvation resistance, repectively. In males, the respective correlations are 0.91, 0.76 and 0.65.

**Figure 1:**
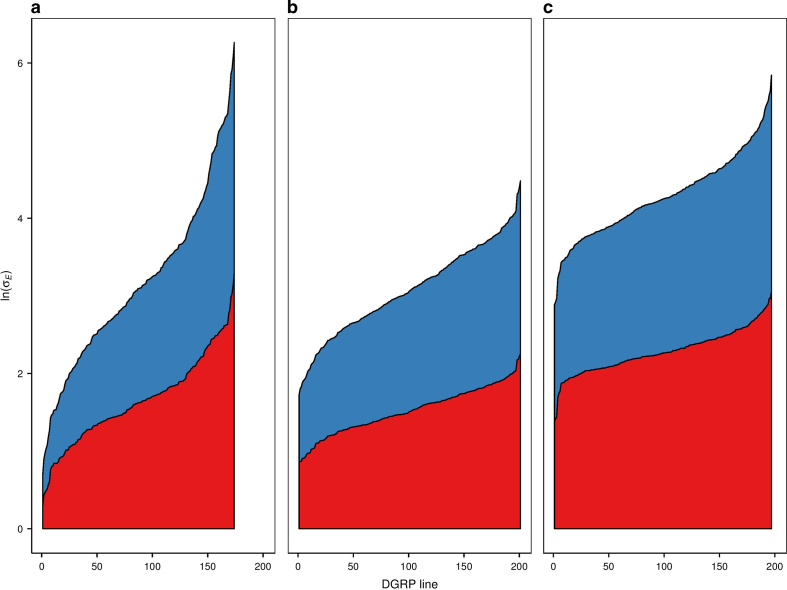
Stackplots showing variation in ln(*σ_E_*) among DGRP lines for females (red) and males (blue) (**a**) Chill coma recovery time. (**b**) Startle response. (**c**) Starvation stress resistance.

We observed considerable variation in ln(*σ_E_*) among lines for all three traits, in both sexes (Fig. 1, Data file S1), indicating that there is genetic variation in micro-environmental plasticity. We next partitioned the variance in ln(*σ_E_*) between sexes and among lines (Table S2). We found significant differences in micro-environmental plasticity between males and females for all three traits. Micro-environmental plasticity is greater for females than males for chill coma recovery and starvation resistance, whereas the reverse is true for startle response. Remarkably, the magnitude of the genetic variance affecting micro-environmental plasticity is very high, with broad sense heritabilities (*H*^2^) of ln(*σ_E_*) of *H*^2^ = 0.75 (chill coma recovery time), *H*^2^ = 0.54 (startle response) and *H*^2^ = 0.36 (starvation resistance). For comparison, the estimates of broad sense heritabilities at the mean level for the same traits and the same dataset were *H*^2^ = 0.37 (chill coma recovery time), *H*^2^ = 0.56 (startle response) and *H*^2^ = 0.58 (starvation resistance)^41^. Thus the broad sense heritability at the variance level is of the same magnitude as that at the level of the mean and, for chill coma recovery time, the heritability of micro-environmental variance is twice as large as that of the mean.

Our observation of significant genetic variation for micro-environmental plasticity of all traits does not depend on the metric used to parameterize micro-environmental plasticity or even whether a natural logarithm transformation is used. Broad sense heritabilities were of similar magnitude regardless of the metric used for all three traits (Table S3). The exception was a lower (but still significant) heritability of micro-environmental plasticity for starvation resistance using *MAD* or ln(*MAD*). We attribute this in part to the strikingly non-normal distribution of both *MAD* and ln(*MAD*) for starvation resistance (Table S1, Fig. S3) and also to the existence of replicates for which the estimate of *MAD* was zero, leading to missing data in the ln(*MAD*) analyses.

Many *Drosophila* quantitative traits exhibit genetic variation in sexual dimorphism for the trait mean; *i.e*., there is a significant sex by line interaction term in the analysis pooled across sexes and the cross-sex genetic correlation (*r_GMF_*) is significantly different from unity^44^. Estimates of *r_GMF_* for the means of the three traits were *σ_E_*  = 1 (startle response), *r_GMF_* = 0.93 (chill coma recovery time) and *r_GMF_* = 0.70 (starvation resistance)^41^. We find similar patterns for the cross-sex genetic correlations of micro-environmental plasticity of the three traits: *r_GMF_*  = 1 (startle response), *r_GMF_* = 0.94 (chill coma recovery time) and *r_GMF_* = 0.58 (starvation resistance) (*P* < 0.00001 for all three traits, Table S4). Therefore, there is considerable genetic variation in sex dimorphism of micro-environmental plasticity for starvation resistance, but not the other two traits.

### Genetic variation in micro-environmental plasticity is not associated with heterozygosity

The DGRP lines are not completely homozygous. Most lines remained segregating for 2% or fewer sites after 20 generations of full sib inbreeding; however, several lines had greater than 20% variants segregating on one or more autosomal arms due to the segregation of large polymorphic inversions^42^. This enabled us to explicitly test the hypothesis that micro-environmental variance may decrease as the heterozygosity increases^45^,^46^. We found no such tendency. None of the correlations of ln(*σ_E_*) with the percentage of segregating sites were significant for any sex or trait (Fig. S4).

### Correlation of micro-environmental plasticity with the mean

**Figure 2:**
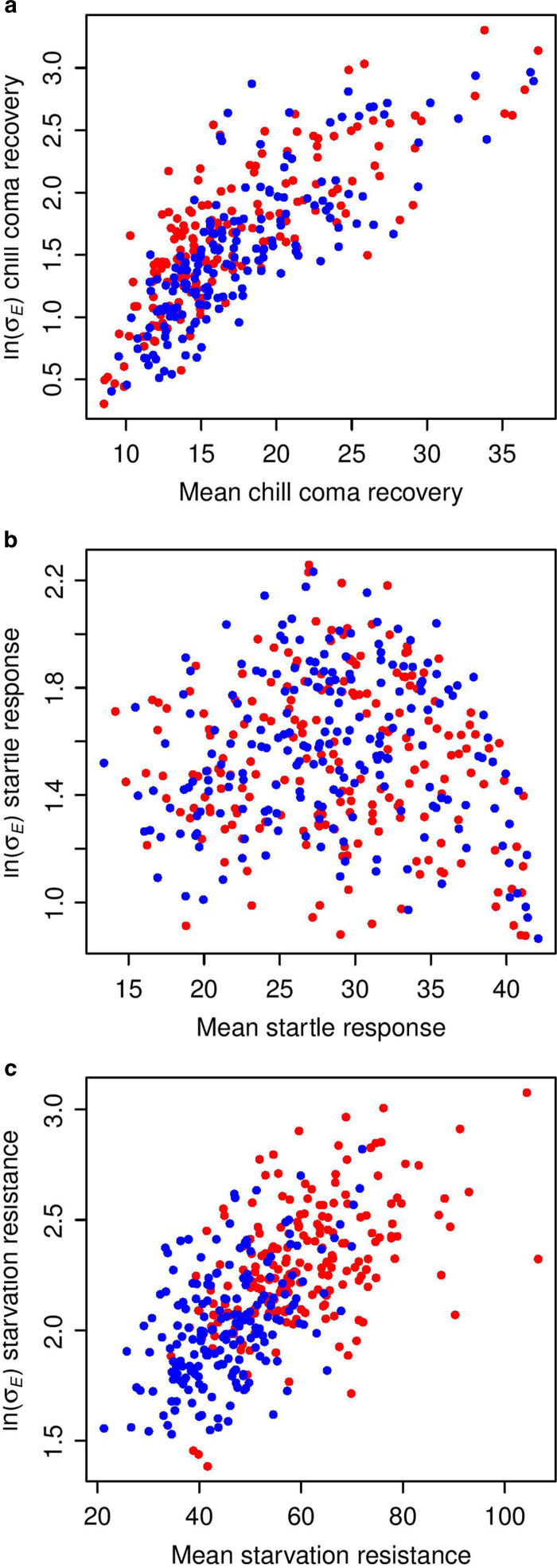
Correlations of mean and micro-environmental variance of three quantitative traits for females (red) and males (blue) (**a**) Chill coma recovery time. The correlations between the mean and micro-environmental variance (*r*_*MV*_) and *r*_*MV *_= 0.79 (*P* < 0.0001) for females and *r*_*MV *_= 0.80 (*P* < 0.0001) for males. (**b**) Startle response. *r*_*MV *_= −0.12 (*P* = 0.10) (females), *r*_*MV *_= −0.03 (*P* = 0.70) (males). (**c**) Starvation resistan**c**e. *r*_*MV *_= 0.50 (*P* < 0.0001) (females), *r*_*MV *_= 0.50 (*P* < 0.0001) (males).

The trajectories of response to natural or artificial selection for the mean and micro-environmental variance depend on whether or not the same variants affecting the mean also affect the micro-environmental variance. Our data on the correlation between line means and micro-environmental variance using ln(*σ_E_*) to parameterize micro-environmental plasticity show all possible relationships (Fig. 2), ranging from high (*r* = 0.80, *P* < 0.00001, chill coma recovery time), to moderate (*r* = 0.50, *P* < 0.00001, starvation resistance) to none (*r* = −0.03, *P* = 0.70, startle response, males).

*CV*_*E*_ is often used to remove any relationship between mean and variance. However, using ln(*CV*_*E*_) to parameterize micro-environmental plasticity did not completely remove its positive correlation with the mean for chill coma recovery, while it introduced artificial negative correlations between the mean and micro-environmental variance of startle response (for which the correlation between the mean and ln(*σ_E_*) is not significant) and starvation resistance (for which there is a positive correlation between the man and ln(*σ_E_*)) (Fig. S5). Thus, while both ln(*σ_E_*) and ln(*CV*_*E*_) indicate variable relationships between trait means and estimates of micro-environmental plasticity, the magnitude and sign of the correlations vary according what metric is used to quantify micro-environmental plasticity. Since the correlations between the mean and micro-environmental plasticity are trait-specific, no single measure of plasticity will result in the same relationship for all traits, so that the choice of the appropriate metric will necessarily depend on the purpose of the analysis. Since responses of micro-environmental plasticity to selection on trait means and the underlying genetic architecture of micro-environmental plasticity depend on genetic correlations between the mean and measures of plasticity that are not corrected for the mean, we prefer the use of ln(*σ_E_*).

### Genetic variation for micro-environmental plasticity is trait-specific

We tested whether the genetic basis of variation for micro-environmental variance is shared between the three traits by partitioning the variance in ln(*σ_E_*) between traits and among lines, separately for males and females (Table S4). We find no significant shared genetic variance across lines; all the genetic variance in ln(*σ_E_*) appears in the trait by line interaction term. Our estimates of the cross-trait genetic correlations (*r_GMF_*) of ln(*σ_E_*) were *r_GMF_* = 0.04 (*P* = 0.60) in females and *r_GMF_* = 0.00 (*P* = 1) in males (Table S4). Estimates of *r_GMF_* for each of the pairs of traits were similarly low and non-significant (Fig. S6). We therefore infer that the genetic basis of variation in micro-environmental plasticity is trait-specific, at least for these three traits.

### Drosophila Hsp90 (Hsp83) is associated with micro-environmental plasticity of startle response

**Figure 3:**
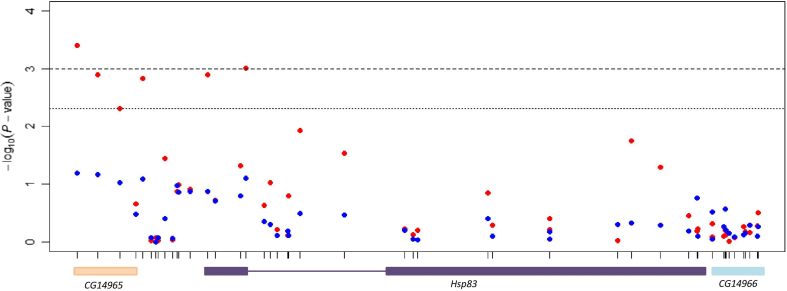
Molecular variants associated with ln(*σ_E_*) of startle response in or near (±1 kb) *Hsp83* for females (red) and males (blue) The physical position of variants is indicted by their relative spacing on the *x*-axis. The *y*-axis gives –log10(*P*-values) for each variant. The dashed line gives the Bonferroni and the dotted line the FDR thresholds corresponding to an experiment-wise *P*-value of 0.05. Two variants met the Bonferroni threshold: *3L*_3191981_SNP (in the exon of *CG14965* and 989 bp upstream of *Hsp83*) and *3L*_3193430_SNP (in the first exon of *Hsp83*). The remaining four variants met the FDR threshold: *3L*_3192162_SNP (in the exon of *CG14965* and 808 bp upstream of *Hsp83*); *3L*_3192350_SNP (in the exon of *CG14965* and 619 bp upstream of *Hsp83*); *3L*_3192548_SNP (58 bp upstream of *CG14965* and 422 bp upstream of *Hsp83*) and *3L*_3193101_SNP (in the first exon of *Hsp83*).

*Hsp90* is thought to affect genetic canalization for many traits in *Drosophila*^47^ and *Arabidopsis*^48^ and has also been associated with micro-environmental variability of *Arabidopsis* hypocotyl elongation in the dark^38^. Therefore, we first tested for associations of 49 common (minor allele frequency, MAF ≥ 0.05) variants (48 for chill coma recovery time) in or within 1 kb of *Hsp83*, the *Drosophila Hsp90* gene, for associations with micro-environmental plasticity of each trait, separately for males and females. We found two significant single nucleotide polymorphisms (SNPs) following a Bonferroni correction and six significant SNPs at a false discovery rate (FDR) of FDR < 0.05 for micro-environmental plasticity of female startle response only (Fig. 3, Fig. S7). The two variants meeting the Bonferroni significant threshold were located in the exon of *CG14965* and 989 bp upstream of *Hsp83* and in the first exon of *Hsp83*. Two of the variants meeting the FDR threshold were in the exon of *CG14965* and upstream of *Hsp83*; one was upstream of both genes; and one was in the first exon of *Hsp83*.

### Genome wide association (GWA) analysis reveals novel candidate genes associated with micro-environmental plasticity

We next performed GWA analyses of ln(*σ_E_*) for the three traits for all common (MAF > 0.05) biallelic variants , using a linear mixed model that accounted for any effects of *Wolbachia* infection, common inversion polymorphisms, and cryptic polygenic relatedness^42^. We did not find any variants associated with ln(*σ_E_*) at a Bonferroni-corrected significance level. Quantile-quantile plots for startle response reveal inflation of *P*-values below *P* < 10^−5^ from a uniform distribution, but not for starvation resistance nor for chill coma recovery time (Fig. S8). We used the *P* < 10^−5^ threshold to report the GWA results for all traits (Data file S2). A total of 75 variants we associated with startle response, of which 32 were intergenic. The remaining variants were associated with 36 unique genes, of which 11 were computationally predicted genes of unknown function. Many of these genes are plausible candidates, as they are involved in development (*form3*, *Src64B*, *ds*, *fru*, *jing*, *IA-2*), including development (*Pde9*, *Src64B, fru*, *jing*) and function (*Dop1R1*, *jing*, *Octbeta3R*) of the nervous system; and behaviors (*Src64B*, *rdgA*, *Dop1R1*, *fru*, *dpr6*, *dpr 8*).

Although there was no signature of enrichment for true positives in the analyses of chill coma recovery time and starvation resistance, we note that two variants (*3L*_2626748_SNP in *Pxn* and *3L*_3533304_SNP in *Eip63E*) were associated with both the mean and micro-environmental variance for chill coma recovery; and one variant (4_655975SNP in *CG1732*) was associated with both the mean and micro-environmental variance for starvation resistance.

## Discussion

We have shown that there is genetic variation in micro-environmental plasticity for two fitness-related traits and an innate behavior in *D. melanogaster*. The broad sense heritabilities were moderate to high, of the same order as the broad sense heritabilities of the trait mean, and robust to the measure used to parameterize micro-environmental plasticity. High broad sense heritabilities of micro-environmental plasticity have been reported for sleep traits and waking activity^36^ and for abdominal bristle number^35^ in this species, and for yeast gene expression^40^. In contrast, broad sense heritabilities of micro-environmental plasticity of *D. melanogaster* sternopleural bristle number (average *H*^2^ = 0.126)^35^ and four morphological and one fitness trait in *A. thaliana* were low (average *H*^2^ = 0.070)^37^, as were narrow sense heritabilities in the studies reviewed in Ref. 22 (average *h*^2^ = 0.035). The high broad sense heritabilities for micro-environmental plasticity might in principle be explained by the two-fold increase in additive genetic variance expected among fully inbred lines relative to the outbred populations from which they were derived^1^, but could also be attributable in part to dominance and/or epistasis^35^. Although the number of studies remains small, there does not appear to be a trend for any difference in the magnitude of genetic variance between traits related to fitness and morphological traits, which are expected to be under directional and stabilizing natural selection, respectively^1^.

The genetic correlation between trait means and their micro-environmental plasticity estimated as ln(*σ_E_*) runs the full gamut from no association (startle response) to highly correlated (chill coma recovery time). The exact relationship between mean and plasticity depends on whether ln(*σ_E_*) or ln(*CV*_*E*_) is used to parameterize plasticity. ln(*σ_E_*) preserves the biological relationship between the mean and micro-environmental plasticity necessary for predicting the correlated responses of micro-environmental plasticity to selection on trait means and pleiotropic effects of variants on both mean and plasticity. Variable relationships between trait means and micro-environmental plasticity are consistent with previous studies in *D. melanogaste*r^36^ and a wide range of species^22^, where mean-environmental variance correlations take on all possible values between high and negative to high and positive. In the latter case, directional selection for an increase in the trait mean will result in more phenotypic variation. This is counter to the uniformity desired in breeding programs, and could possibly account for the high environmental variance generally observed for traits associated with fitness. Similarly, high positive or negative correlations between mean and environmental variance for traits under constant stabilizing selection will impose a limit to selection for zero environmental variance. Production traits for which there is a low correlation between the mean and environmental variance are of most interest to applied breeding programs, since response to selection for both the mean and uniformity can increase economic value^22^,^23^,^24^.

We assessed genetic variation in micro-environmental plasticity separately for males and females, and for three quantitative traits, which enabled us to assess whether the genetic architecture of micro-environmental plasticity is the same in both sexes, or is common to multiple traits. Similar to the genetic architecture of the mean for these traits^41^, as well as other quantitative traits^44^, there is variation in the extent to which the genetic basis of micro-environmental plasticity is sex-specific. In contrast, the genetic basis of micro-environmental plasticity is clearly trait-specific, at least for the three traits studied here. It has been proposed that *Hsp90* is a capacitor of phenotypic variation, buffering both genetic and micro-environmental variation in *Drosophila* and *Arabidopsis*^38^,^47^,^48^. While we have no evidence for a common genetic basis for micro-environmental plasticity across traits, variants in *Drosophila Hsp90* (*Hsp83*) are indeed associated with micro-environmental in female startle response. We performed GWA analyses to identify common genetic variants associated with micro-environmental plasticity, and identified several plausible candidate genes that can be investigated in the future using the genetic resources available in *D. melanogaster*. However, the power of these analyses was low because the sampling error of ln(*σ_E_*) is much higher than that of trait means^22^.

The empirical study of the genetic basis of micro-environmental plasticity is in its infancy, with most previous studies aiming simply to document its existence. We have definitively shown that there is substantial genetic variance in micro-environmental plasticity, motivating future well-powered experiments to accurately estimate micro-environmental variance and map the variants associated with this variation. Open questions include the relative contributions of additive, dominance and epistasis to the genetic variance of micro-environmental variance; the relationship between the genetic basis of micro-environmental plasticity, within-individual plasticity (fluctuating asymmetry, special environmental variance) and genotype by environment interaction (macro-environmental variance); and the nature of the evolutionary forces maintaining segregating variation for micro-environmental plasticity. The converse of plasticity is canalization, which also raises the question of whether environmental canalization and genetic canalization (epistasis) have the same genetic basis^49^. Model organisms that can be inbred to homozygosity will play a key role in addressing these questions.

## Methods

### *Drosophila* lines and phenotypic data

The DGRP consists of 205 inbred lines obtained by 20 generations of full-sib mating from isofemale lines collected from the Raleigh, NC, USA population, and which have full genome sequences^42^. All flies were reared under standard culture conditions (cornmeal-molasses-agar-medium, 25 °C, 60–75% relative humidity, 12-hr light-dark cycle).

We quantified resistance to starvation stress for 197 DGRP lines^42^ by placing 10 same-sex, two day-old flies in culture vials containing non-nutritive medium (1.5% agar and 5 ml water), and scoring survival every eight hours until all flies were dead^50^. There were five replicate vials/sex/line (total *N* = 19,361; female *N* = 9,672; male *N* = 9,689). We measured time to recover from a chill-induced coma for 174 DGRP lnes by transferring three to seven day-old flies without anesthesia to empty vials, and placing them on ice for three hours. We then transferred the flies to room temperature, and recorded the time it took for each individual to right itself and stand on its legs^51^. There were two replicates of 50 flies/sex/line (total *N* = 34,653; female *N* = 17,382; male *N* = 17,271). We assessed startle response for 201 DGRP lines^44^ by placing single three to seven day-old adult flies, collected under CO_2_ exposure, into vials containing 5 ml culture medium, and leaving them overnight to acclimate to their new environment. On the next day, between 8am and 12pm (2 – 6 hours after lights on), each fly was subjected to a gentle mechanical disturbance, and the total amount of time the fly was active in the 45 seconds immediately following the disturbance was recorded. There were two replicates of 20 flies/sex/line (total *N* = 16,214; female *N* = 8,114; male *N* = 8,100).

### Genetic variance of micro-environmental plasticity

We tested heterogeneity of within line variances among lines using the Brown-Forsythe and Levene’s tests. Both tests use a transformation of the response variable as 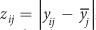, where *y*_*ij*_ is the *i*^th^ individual of the *j*^th^ line, and 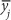 is the median or the mean of the *j*^th^ line for the Brown-Forsythe test and Levene’s test, respectively.

We estimated the error mean square separately for each line, trait, sex and replicate by fitting linear models which included only the intercept (*Y* = *μ* + *E*, where *Y* is the phenotypic value of the trait, *μ* is the overall mean and *E* is the within-replicate, *i.e*., micro-environmental, random error). We estimated the micro-environmental standard deviation, *σ*_*E*_, as the square root of the mean square errors, giving two records (five for starvation resistance) for each replicate and line. We used the natural logarithm of *σ*_*E*_ as our metric of micro-environmental variance. We also estimated *CV*_*E*_ as 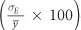, where *ỹ* is the phenotypic mean. We estimated *MAD* as median (|*y*_*ij*_ − *ỹ*_*j*_ |), where *y*_*ij*_ is the *i*^th^ individual of the *j*^th^ line and *ỹ*_j_ is the median of the *j*^th^ line. We tested for approximate normality of all metrics using Pearson’s χ^2^ goodness-of-fit test.

We assessed the genetic variance of ln(*σ*_*E*_) pooled across sexes using a mixed model factorial ANOVA model of form *Y* = *μ* + *S* + *L* + *S* × *L* + *E*, where *Y* is ln(*σ*_*E*_), *μ* is the overall mean, *S* is the cross-classified fixed effect of sex, *L* is the random effect of line, *S* × *L* is the random effect of the interaction between sex and line and *E* is the between-replicates random error. We estimated the broad sense heritability of micro-environmental heterogeneity as 
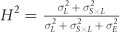
 , where *σ*^2^ denotes the variance component of the term corresponding to the subscript. We computed cross-sex genetic correlations of ln(*σ*_*E*_) as 
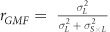
. We performed the same analyses for all other metrics of micro-environmental plasticity.

We also performed reduced ANOVAs, separately for each sex, of form *Y* = *μ* + *L* + *E*, where *Y* is ln(*σ*_*E*_), *μ* is the overall mean, *L* is the random effect of the line and *E* is the between-replicate random error. The broad sense heritability was then estimated as 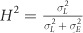. The significance of the main effects and interactions were tested using F-tests. We performed the same analyses for all other metrics of micro-environmental plasticity.

To test the effect of trait on micro-environmental plasticity, we performed mixed model factorial ANOVAs of form *Y* = *μ* + *T* + *L* + *T* × *L* + *E*, separately for males and females, where *Y* is ln(*σ*_*E*_), *μ* is the overall mean, *T* is the fixed effect of trait, *L* is the random effect of the trait, *T*×*L* is the random effect of the interaction between trait and line and *E* is the random error, separately for males and females. The significance of the main effects and interactions were tested using F-tests. We computed cross-trait genetic correlations of ln(*σ*_*E*_) as 
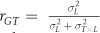
.

We computed the Pearson product moment correlations of ln(*σ*_*E*_) with trait means and % segregating sites.

### GWA analyses of micro-environmental plasticity

We estimated the error mean square for each trait, line and sex, pooled across replicates using ANOVA models of form *Y* = *μ* + *R* + *E*, where *Y* is the phenotypic value of the trait, *R* is the random effect of the replicate and *E* is the random error. We computed the residual standard deviation pooled across replicates, *σ*_*ER*_, as the square root of the mean square error, and used ln(*σ*_*ER*_) as the metric of environmental variance of each line. We then performed association analyses for each trait for all segregating biallelic variants with minor allele frequencies ≥0.05 and for which the Phred scaled variant quality was greater than 500 and the genotype call rate was ≥0.8. We used a mixed model accounting for the relationship among lines, the effect of inversions and *Wolbachia* infection as implemented in the DGRP website^42^. Annotation was performed according to FlyBase^52^. We first tested the effects of common variants in or near (±1,000 bp) the candidate gene *Hsp83* (*Drosophila Hsp90*), and then performed a full GWA analysis.

## Author Contributions

F.M., P.S, D.S., C.M. and T.F. C.M. designed the experiment. F.M. performed the analyses. F.M. and T.F.C.M. wrote the manuscript.

## Additional Information

**How to cite this article**: Morgante, F. *et al.* Genetic Architecture of Micro-Environmental Plasticity in Drosophila melanogaster. *Sci. Rep*. **5**, 09785 doi: 10.1038/srep09785 (2015).

## Supplementary Material

Supplementary Information

Supplementary Data file S1

Supplementary Data file S2

## Figures and Tables

**Table 1:** Tests for heterogeneity of within-line variances (a) Brown-Forsythe test. (b) Levene’s test.

(a)				
Analysis	Trait	df	F	P-value
Females	Chill coma recovery	173	21.74	<0.0001
	Startle response	200	9.496	<0.0001
	Starvation resistance	196	5.316	<0.0001
Males	Chill coma recovery	173	17.981	<0.0001
	Startle response	200	8.051	<0.0001
	Starvation resistance	196	4.774	<0.0001
**(b)**				
Analysis	Trait	df	F	P-value
Females	Chill coma recovery	173	34.704	<0.0001
	Startle response	200	11.193	<0.0001
	Starvation resistance	196	6.26	<0.0001
Males	Chill coma recovery	173	29.26	<0.0001
	Startle response	200	9.56	<0.0001
	Starvation resistance	196	5.928	<0.0001
