# Supplementary material for: Genetic Architecture of Micro-Environmental Plasticity in Drosophila melanogaster
Source: Sci Rep. 2015 May 6;5:9785. doi: 10.1038/srep09785 (PMC4421828; doi:10.1038/srep09785)
Supplement: Supplementary Information [file srep09785-s1.pdf]

Supplementary information

Genetic Architecture of Micro-Environmental Plasticity in *Drosophila melanogaster*

Fabio Morgante, Peter Sørensen, Daniel A. Sorensen,

Christian Maltecca and Trudy F. C. Mackay

Fig. S1| Density plots showing skewed distributions of σ_E and ln(σ_E) for females (red) and males (blue). (**a, b**) Chill coma recovery time. (**c, d**) Startle response. (**e, f**) Starvation stress resistance.

Fig. S1

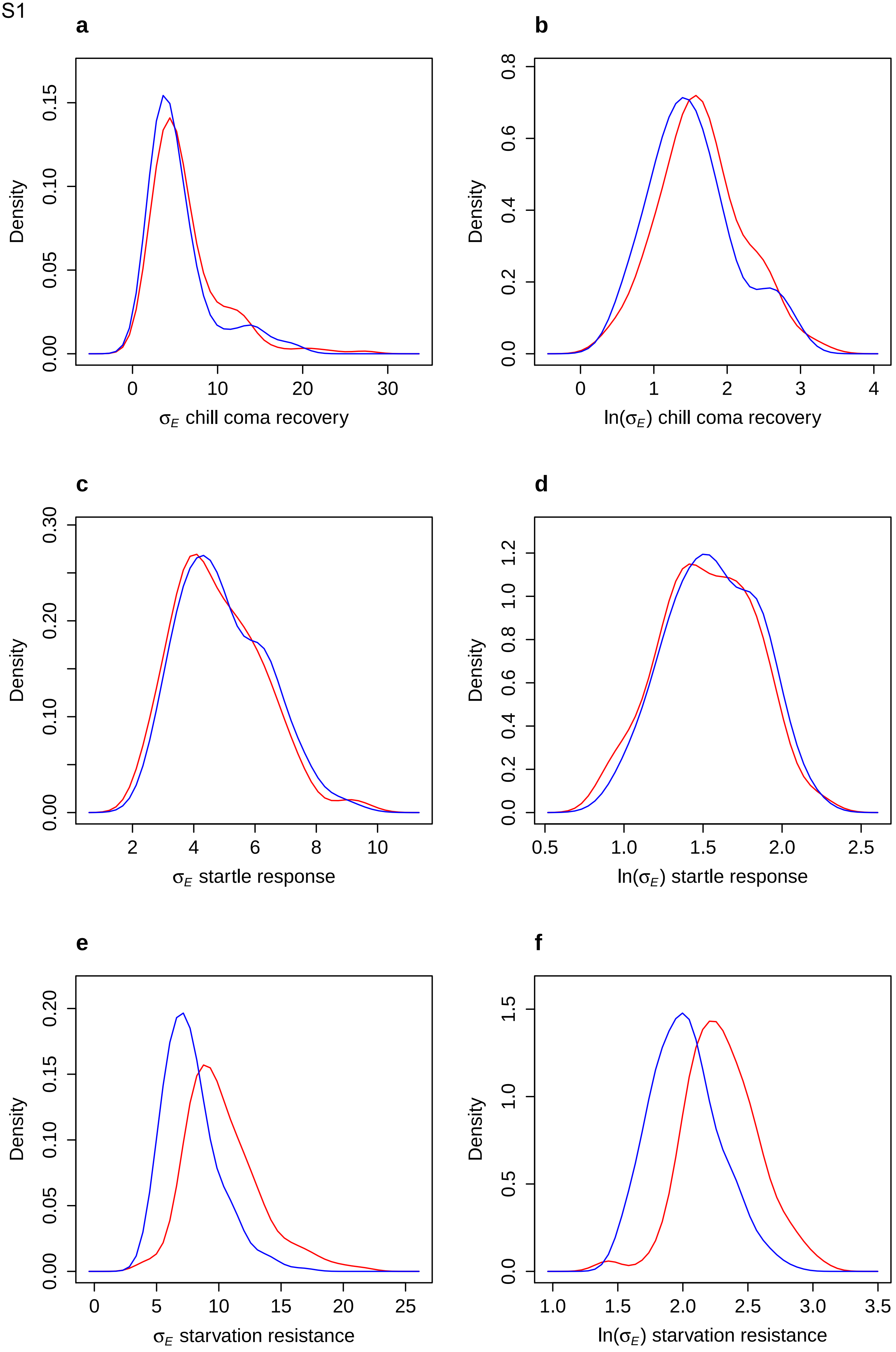

a
Density
0.00
0.05
0.10
0.15
0
10
20
30
σE chill coma recovery
b
Density
0.0
0.2
0.4
0.6
0.8
0
1
2
3
4
ln(σE) chill coma recovery
c
Density
0.00
0.10
0.20
0.30
2
4
6
8
10
σE startle response
d
Density
0.0
0.2
0.4
0.6
0.8
1.0
1.2
0.5
1.0
1.5
2.0
2.5
ln(σE) startle response
e
Density
0.00
0.05
0.10
0.15
0.20
0
5
10
15
20
25
σE starvation resistance
f
Density
0.0
0.5
1.0
1.5
1.0
1.5
2.0
2.5
3.0
3.5
ln(σE) starvation resistance

Fig. S2| Density plots showing skewed distributions of CV_E and ln(CV_E) for females (red) and males (blue). (**a, b**) Chill coma recovery time. (**c, d**) Startle response. (**e, f**) Starvation stress resistance.

Fig. S2

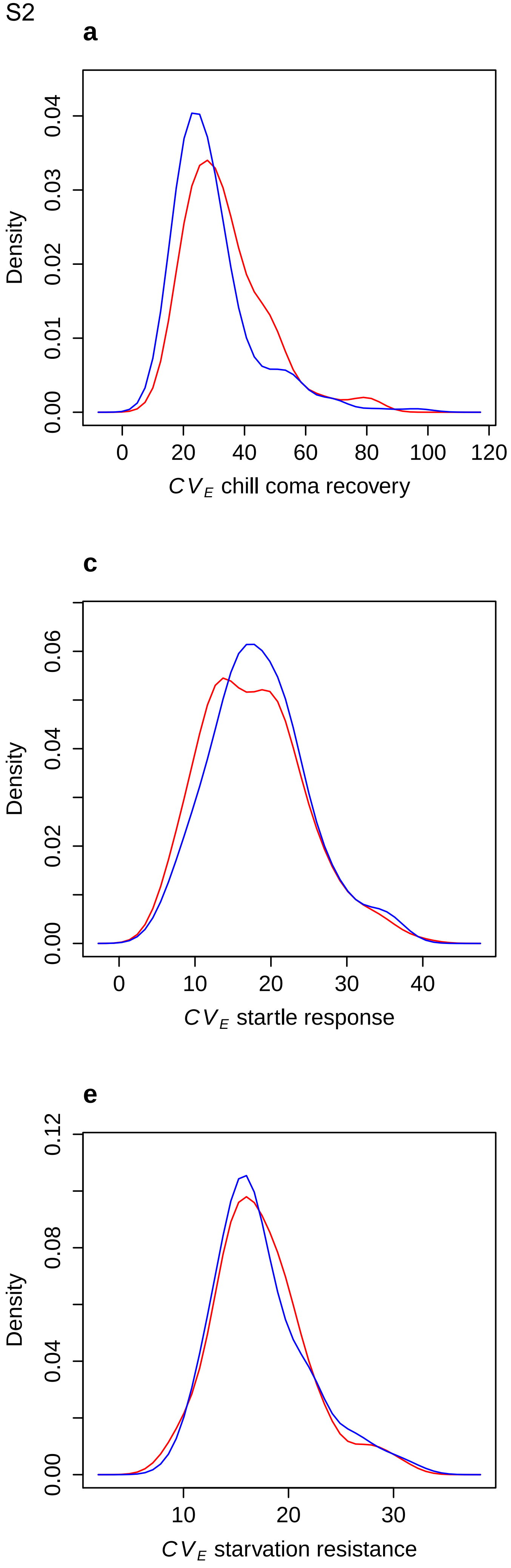
a
Density
0.00
0.01
0.02
0.03
0.04
0
20
40
60
80
100
120
CV_E chill coma recovery
b
Density
0.0
0.2
0.4
0.6
0.8
1.0
1.2
2.0
2.5
3.0
3.5
4.0
4.5
5.0
ln(CV_E) chill coma recovery
c
Density
0.00
0.02
0.04
0.06
0
10
20
30
40
CV_E startle response
d
Density
0.0
0.2
0.4
0.6
0.8
1.0
1.2
1.5
2.0
2.5
3.0
3.5
4.0
ln(CV_E) startle response
e
Density
0.00
0.04
0.08
0.12
10
20
30
CV_E starvation resistance
e
Density
0.0
0.5
1.0
1.5
2.0
2.5
3.0
3.5
ln(CV_E) starvation resistance

Fig. S3| Density plots showing skewed distributions of *MAD* and ln(*MAD*) for females (red) and males (blue). (**a, b**) Chill coma recovery time. (**c, d**) Startle response. (**e, f**) Starvation stress resistance.

Fig. S3

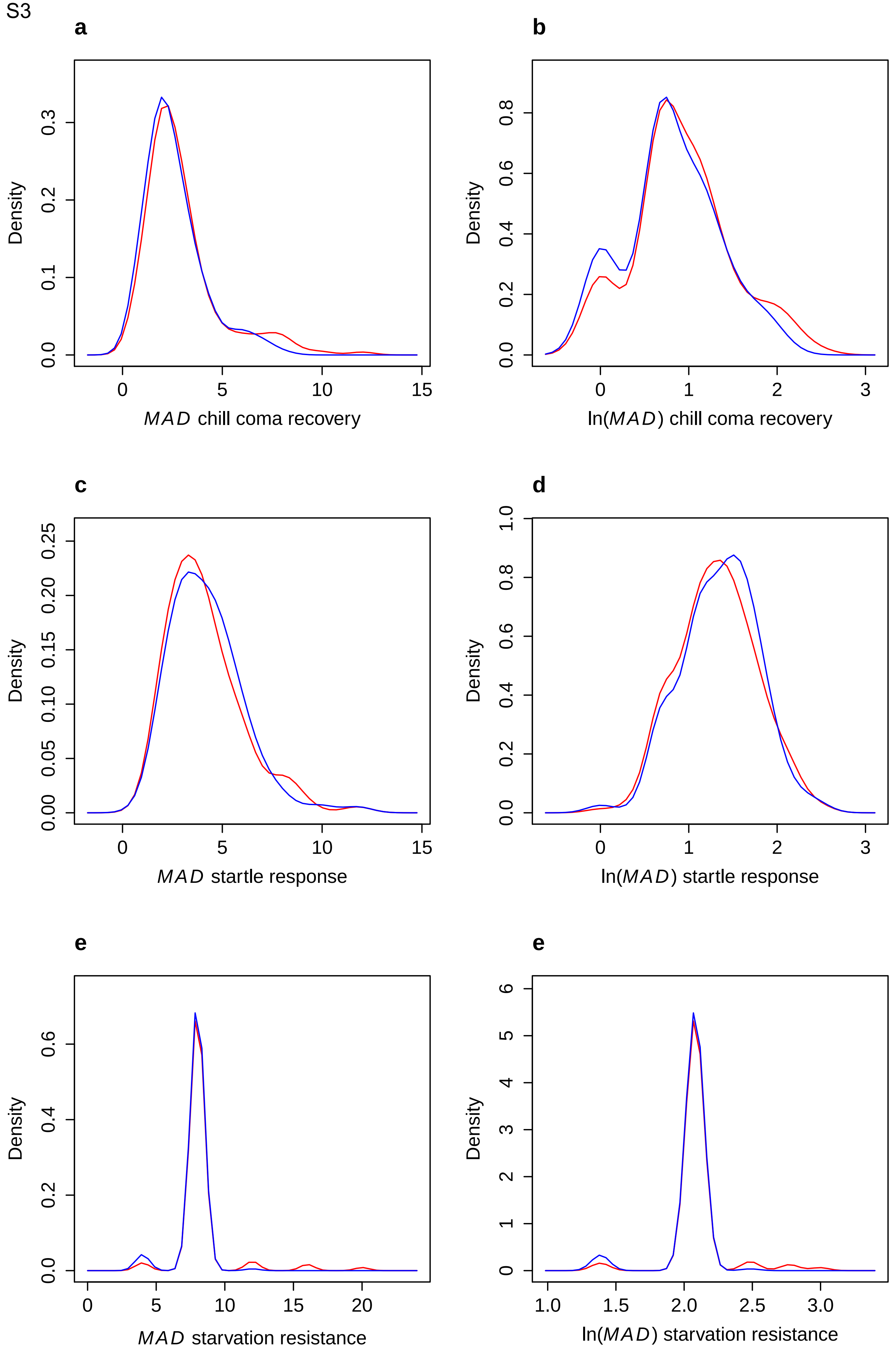
a
Density
MAD chill coma recovery
b
Density
ln(MAD) chill coma recovery
c
Density
MAD startle response
d
Density
ln(MAD) startle response
e
Density
MAD starvation resistance
e
Density
ln(MAD) starvation resistance

Fig. S4| Correlations of % segregating sites and micro-environmental variances of three quantitative traits for females (red) and males (blue). (**a**) Chill coma recovery time. The correlations between the % segregating sites and micro-environmental variance (r_{MSS}) are r_{MSS} = -0.09 (P = 0.26) (females) and r_{MSS} = -0.08 (P = 0.30) (males). (**b**) Startle response. r_{MSS} = 0.02 (P = 0.80) (females), r_{MSS} = -0.07 (P = 0.34) (males). (**c**) Starvation resistance. r_{MSS} = 0.10 (P = 0.15) (females), r_{MSS} = 0.11 (P = 0.13) (males).

Fig. S4

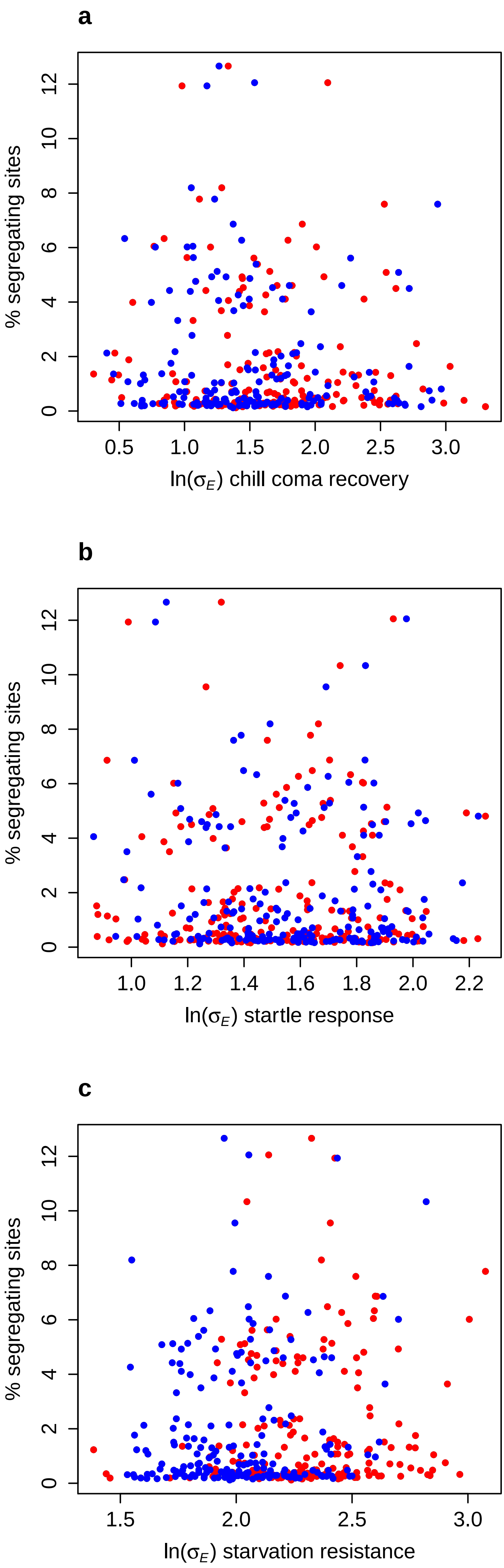
a
% segregating sites
0
2
4
6
8
10
12
0.5
1.0
1.5
2.0
2.5
3.0
ln(σ_E) chill coma recovery
b
% segregating sites
0
2
4
6
8
10
12
1.0
1.2
1.4
1.6
1.8
2.0
2.2
ln(σ_E) startle response
c
% segregating sites
0
2
4
6
8
10
12
1.5
2.0
2.5
3.0
ln(σ_E) starvation resistance

Fig. S5| Comparison of correlations of trait means and micro-environmental plasticity for three quantitative traits using two different metrics to parameterize micro-environmental plasticity, ln(σ_E) and ln(CV_E)). Red points indicate females and blue points indicate males. (**a**) Mean vs ln(σ_E) for chill coma recovery time [r_{MV} = 0.79 (P < 0.0001) for females and r_{MV} = 0.80 (P < 0.0001) for males]. (**b**) Mean vs ln(CV_E) for chill coma recovery time [r_{MV} = 0.40 (P < 0.0001) for females and r_{MV} = 0.48 (P < 0.0001) for males]. (**c**) Mean vs ln(σ_E) for startle response [r_{MV} = -0.12 (P = 0.10) for females and r_{MV} = -0.03 (P = 0.70) for males]. (**d**) Mean vs ln(CV_E) for startle response [r_{MV} = -0.68 (P < 0.0001) for females and r_{MV} = -0.66 (P < 0.0001) for males]. (**e**) Mean vs ln(σ_E) for starvation resistance [r_{MV} = 0.50 (P < 0.0001) for females and r_{MV} = 0.50 (P < 0.0001) for males]. (**f**) Mean vs ln(CV_E) for starvation resistance [r_{MV} = -0.27 (P < 0.0001) for females and r_{MV} = -0.33 (P < 0.0001) for males].

Fig. S5

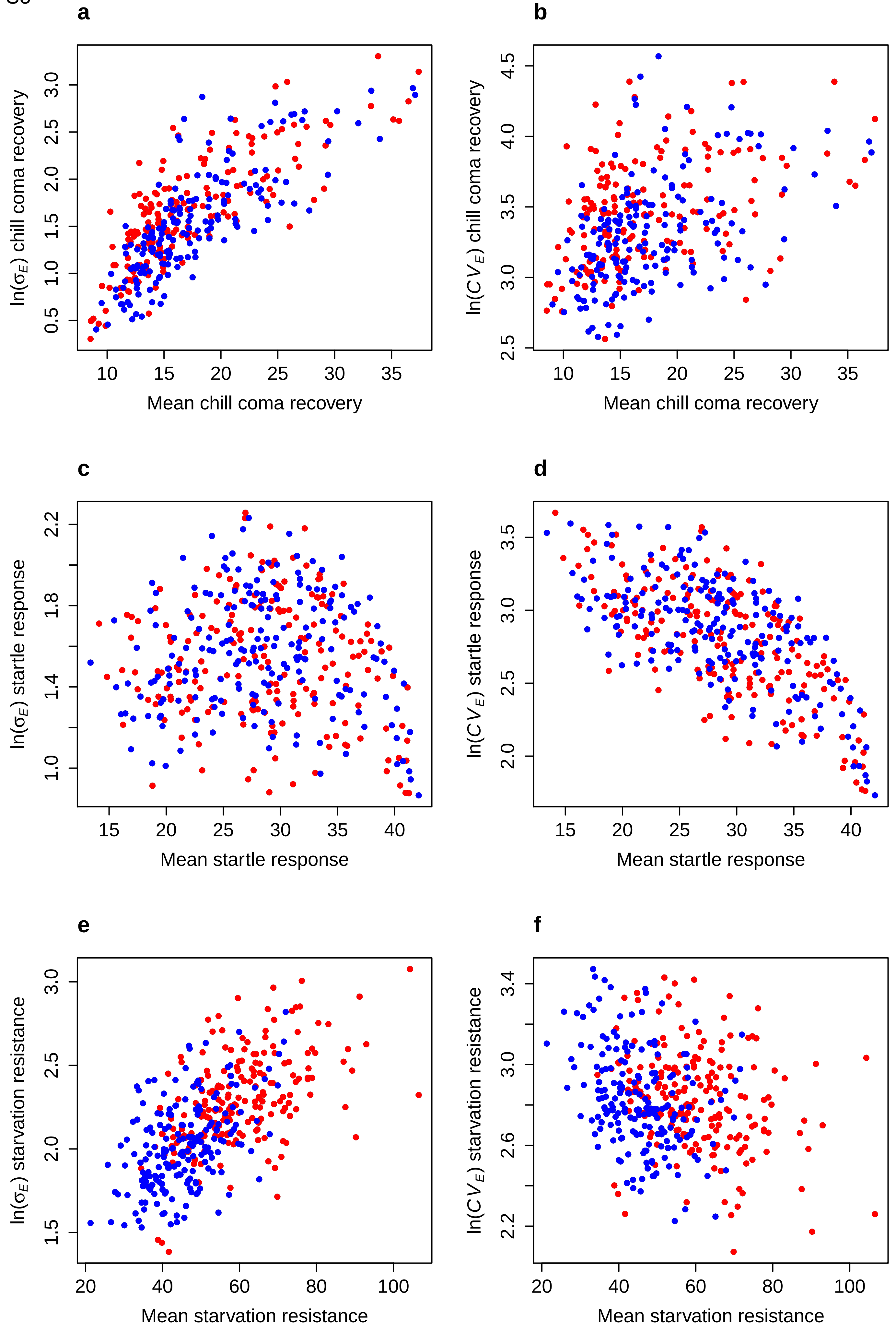

a
ln(σ_E) chill coma recovery
Mean chill coma recovery
b
ln(CV_E) chill coma recovery
Mean chill coma recovery
c
ln(σ_E) startle response
Mean startle response
d
ln(CV_E) startle response
Mean startle response
e
ln(σ_E) starvation resistance
Mean starvation resistance
f
ln(CV_E) starvation resistance
Mean starvation resistance

Fig. S6| Correlations of micro-environmental variance between pairs of quantitative traits for females (red) and males (blue). (**a**) Chill coma recovery time and startle response. *r* = -0.02 (*P* = 0.81) (females), *r* = -0.07 (*P* = 0.39) (males). (**b**) Chill coma recovery and starvation resistance. *r* = 0.08 (*P* = 0.29) (females), *r* = 0.03 (*P* = 0.71) (males). (**c**) startle response and starvation resistance. *r* = 0.06 (*P* = 0.40) (females), *r* = 0.005 (*P* = 0.94) (males).

Fig. S6

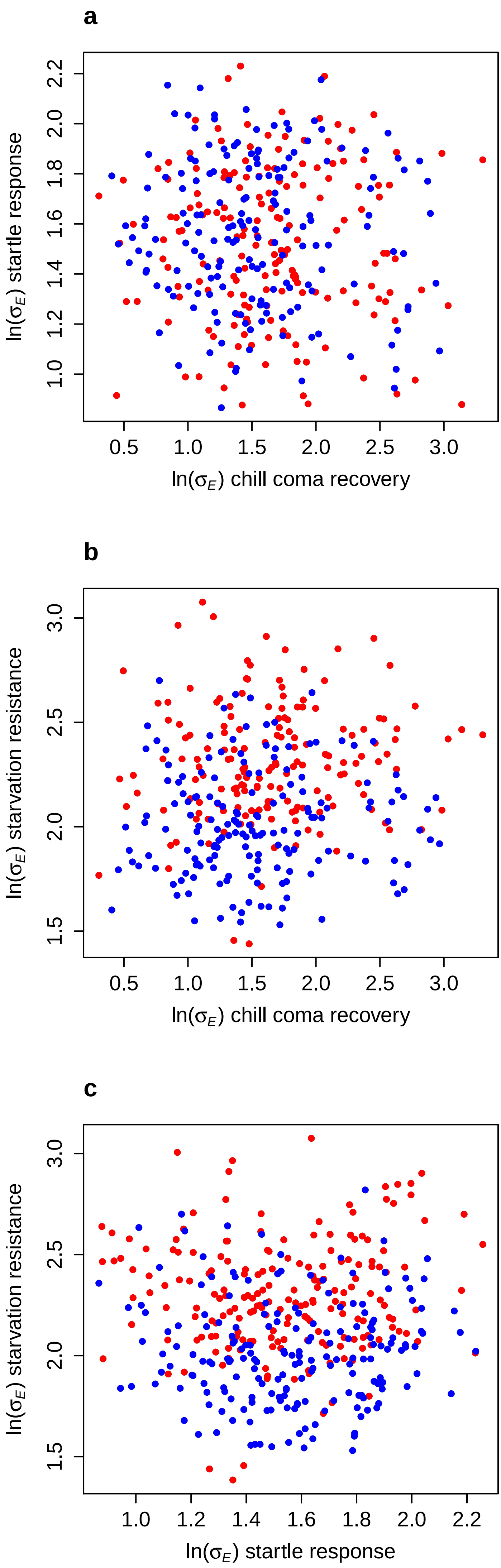
a
ln(σE) startle response
2.2
2.0
1.8
1.6
1.4
1.2
1.0
0.5
1.0
1.5
2.0
2.5
3.0
ln(σE) chill coma recovery
b
ln(σE) starvation resistance
3.0
2.5
2.0
1.5
0.5
1.0
1.5
2.0
2.5
3.0
ln(σE) chill coma recovery
c
ln(σE) starvation resistance
3.0
2.5
2.0
1.5
1.0
1.2
1.4
1.6
1.8
2.0
2.2
ln(σE) startle response

Fig. S7| Molecular variants associated with ln(σ_E) in or near (± 1 kb) *Hsp83* for females (red) and males (blue). The *y*-axis gives –log10(*P*-values) for each variant. The dashed line gives the Bonferroni threshold corresponding to an experiment-wise *P*-value of 0.05. (**a**) Chill coma recovery time. (**b**) Starvation resistance.

Fig. S7

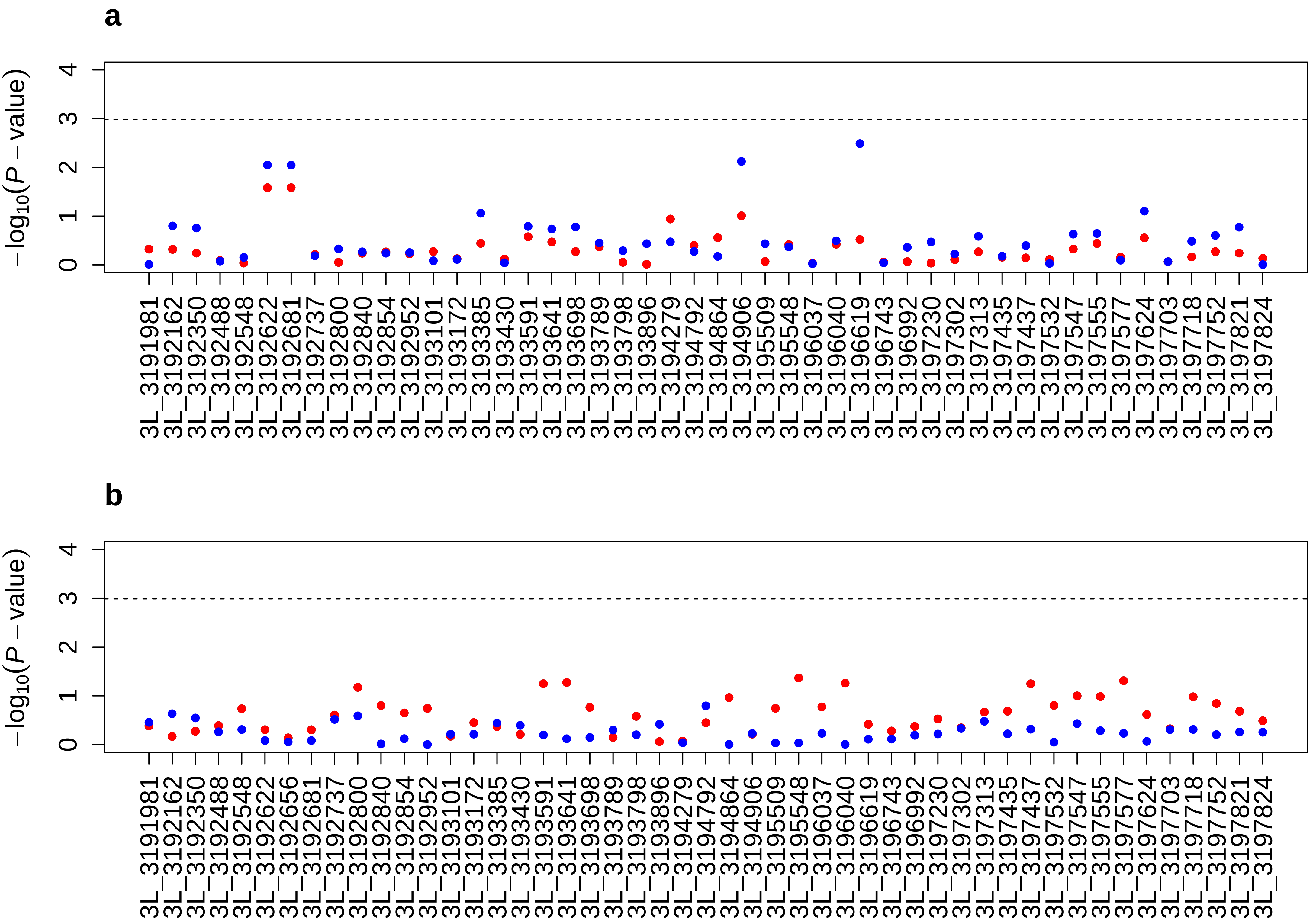
a
−log10(P − value)
0
1
2
3
4
3L_3191981
3L_3192162
3L_3192350
3L_3192488
3L_3192548
3L_3192622
3L_3192681
3L_3192737
3L_3192800
3L_3192840
3L_3192854
3L_3192952
3L_3193101
3L_3193172
3L_3193385
3L_3193430
3L_3193591
3L_3193641
3L_3193698
3L_3193789
3L_3193798
3L_3193896
3L_3194279
3L_3194792
3L_3194864
3L_3194906
3L_3195509
3L_3195548
3L_3196037
3L_3196040
3L_3196619
3L_3196743
3L_3196992
3L_3197230
3L_3197302
3L_3197313
3L_3197435
3L_3197437
3L_3197532
3L_3197547
3L_3197555
3L_3197577
3L_3197624
3L_3197703
3L_3197718
3L_3197752
3L_3197821
3L_3197824
b
−log10(P − value)
0
1
2
3
4
3L_3191981
3L_3192162
3L_3192350
3L_3192488
3L_3192548
3L_3192622
3L_3192656
3L_3192681
3L_3192737
3L_3192800
3L_3192840
3L_3192854
3L_3192952
3L_3193101
3L_3193172
3L_3193385
3L_3193430
3L_3193591
3L_3193641
3L_3193698
3L_3193789
3L_3193798
3L_3193896
3L_3194279
3L_3194792
3L_3194864
3L_3194906
3L_3195509
3L_3195548
3L_3196037
3L_3196040
3L_3196619
3L_3196743
3L_3196992
3L_3197230
3L_3197302
3L_3197313
3L_3197435
3L_3197437
3L_3197532
3L_3197547
3L_3197555
3L_3197577
3L_3197624
3L_3197703
3L_3197718
3L_3197752
3L_3197821
3L_3197824

Fig. S8| Q-Q plots of –$\log_{10}$(*P*-values) from GWA analyses of ln(σ_E) (*y*-axis) and expected under a uniform distribution (*x*-axis). (**a**) Chill coma recovery time, females, (**b**) Chill coma recovery time, males. (**c**) Startle response, females. (**d**) Startle response, males. (**e**) Starvation resistance, females. (**f**) Starvation resistance, males.

Fig. S8

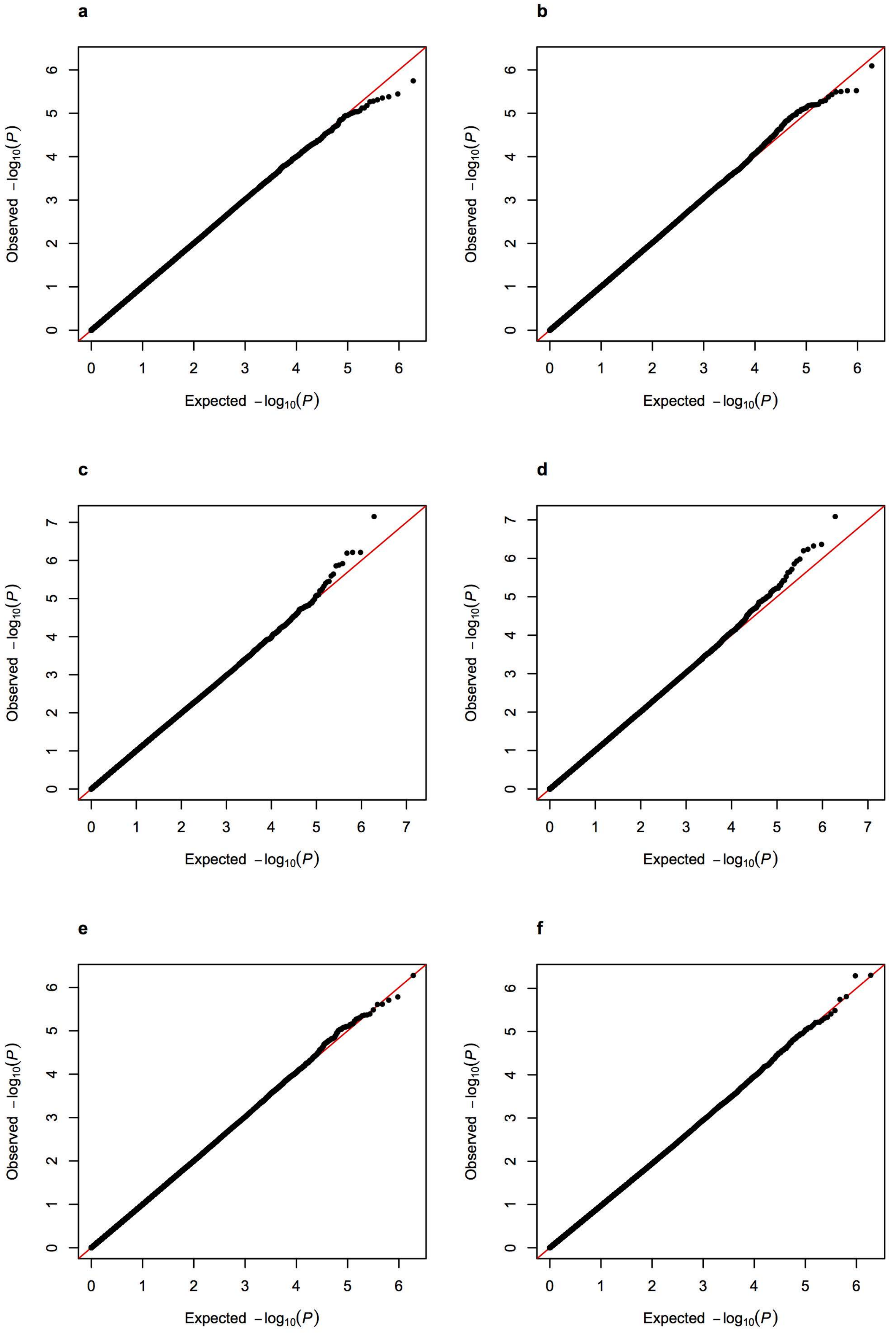

Table S1| Pearson's χ^2 tests for normality.

Metric	Analysis	Trait	P	*P*-value
$\boldsymbol{\sigma_E}$	Females	Chill coma recovery	82.368	<0.0001
		Startle response	20.338	0.120
		Starvation resistance	47.817	<0.0001
	Males	Chill coma recovery	108.851	<0.0001
		Startle response	26.597	0.022
		Starvation resistance	41.777	0.0001
ln(σ_E)	Females	Chill coma recovery	18.553	0.138
		Startle response	12.050	0.602
		Starvation resistance	22.792	0.064
	Males	Chill coma recovery	23.885	0.032
		Startle response	18.139	0.200
		Starvation resistance	20.548	0.114
CV_E	Females	Chill coma recovery	46.690	<0.0001
		Startle response	24.229	0.043
		Starvation resistance	22.964	0.061
	Males	Chill coma recovery	84.575	<0.0001
		Startle response	21.184	0.097
		Starvation resistance	49.716	<0.0001
ln(CV_E)	Females	Chill coma recovery	14.506	0.339
		Startle response	21.184	0.097
		Starvation resistance	18.822	0.172
	Males	Chill coma recovery	23.333	0.038
		Startle response	15.940	0.317
		Starvation resistance	24.518	0.040
MAD	Females	Chill coma recovery	418.000	<0.0001
		Startle response	161.582	<0.0001
		Starvation resistance	2286.467	<0.0001
	Males	Chill coma recovery	480.713	<0.0001
		Startle response	171.562	<0.0001
		Starvation resistance	1889.513	<0.0001
ln(*MAD*)	Females	Chill coma recovery	418.000	<0.0001
		Startle response	162.090	<0.0001
		Starvation resistance	197.000	<0.0001
	Males	Chill coma recovery	475.195	<0.0001
		Startle response	169.194	<0.0001
		Starvation resistance	197.000	<0.0001

Table S2| Quantitative genetic analysis of variance in micro-environmental plasticity for three traits, pooled across sexes. df: degrees of freedom; MS: Mean squares; F: F-ratio test; σ^2 (s.e.): variance component (standard error); H^2: broad sense heritability; r_{GMF}: Cross-sex genetic correlation. Variance components given as -0.000 were negative.

Trait	Analysis	Source	df	MS	F	*P*-value	σ^2 (s.e.)	H^2	r_{GMF}
Chill coma recovery	Sexes pooled	Sex	1	2.78	23.80	< 0.0001	N/A	0.75	0.94
		Line	173	1.15	9.81	< 0.0001	0.258 (0.031)		
		Sex×Line	173	0.12	1.33	0.0127	0.015 (0.007)		
		Error	348	0.09			0.088 (0.007)		
	Females	Line	173	0.65	7.70	< 0.0001	0.281 (0.035)	0.77	
		Error	174	0.08			0.084 (0.009)		
	Males	Line	173	0.62	6.79	< 0.0001	0.263 (0.034)	0.74	
		Error	174	0.09			0.091 (0.010)		
Startle response	Sexes pooled	Sex	1	0.31	7.24	0.0077	N/A	0.54	1.00
		Line	200	0.30	6.94	< 0.0001	0.064 (0.007)		
		Sex×Line	200	0.04	0.78	0.9735	-0.000 (0.003)		
		Error	402	0.05			0.055 (0.004)		
	Females	Line	200	0.17	3.08	< 0.0001	0.059 (0.009)	0.51	
		Error	201	0.06			0.057 (0.006)		
	Males	Line	200	0.16	3.17	< 0.0001	0.056 (0.009)	0.52	
		Error	201	0.05			0.052 (0.005)		
Starvation resistance	Sexes pooled	Sex	1	35.78	183.13	< 0.0001	N/A	0.36	0.58
		Line	196	0.48	2.48	< 0.0001	0.029 (0.005)		
		Sex×Line	196	0.19	2.22	< 0.0001	0.021 (0.004)		
		Error	1,571	0.09			0.088 (0.003)		
	Females	Line	196	0.36	3.83	< 0.0001	0.054 (0.007)	0.36	
		Error	786	0.09			0.095 (0.005)		
	Males	Line	196	0.31	3.84	< 0.0001	0.047 (0.006)	0.36	
		Error	785	0.08			0.082 (0.004)		

Table S3| Quantitative genetic analyses of variance in micro-environmental plasticity using different metrics to parameterise micro-environmental plasticity. (**a**) Analyses of untransformed data. (**b**) Analyses of ln-transformed data. df: degrees of freedom; F: F-ratio test; σ^2: variance component; H^2: broad sense heritability. Variance components given as -0.000 were negative.

**a**			***CV_E***				***MAD***				**σ_E**			
**Trait**	**Analysis**	**Source**	**F**	***P*-value**	**σ^2**	**H^2**	**F**	***P*-value**	**σ^2**	**H^2**	**F**	***P*-value**	**σ^2**	**H^2**
Chill coma recovery	Sexes pooled	Sex	36.97	<0.0001	N/A		12.42	0.0005	N/A		12.81	0.0004	N/A	
		Line	5.86	<0.0001	111.440	0.61	6.60	<0.0001	1.888	0.72	8.03	<0.0001	11.487	0.73
		Sex×Line	1.21	0.071	7.923		1.64	<0.0001	0.262		1.41	0.004	0.956	
		Error			75.859				0.825				4.624	
	Females	Line	4.31	<0.0001	124.056	0.62	10.46	<0.0001	2.801	0.83	7.82	<0.0001	14.144	0.77
		Error			74.921				0.592				4.139	
	Males	Line	3.99	<0.0001	114.669	0.60	3.84	<0.0001	1.500	0.59	5.22	<0.0001	10.772	0.68
		Error			76.796				1.057				5.109	
Startle response	Sexes pooled	Sex	6.70	0.010	N/A		10.68	0.001	N/A		6.23	0.013	N/A	
		Line	10.12	<0.0001	35.312	0.47	5.23	<0.0001	0.920	0.43	7.59	<0.0001	1.530	0.51
		Sex×Line	0.5	1	-0.000		0.8	0.960	-0.000		0.72	0.995	-0.000	
		Error			31.165				1.085				1.286	
	Females	Line	2.51	<0.0001	26.588	0.43	2.27	<0.0001	0.708	0.39	3.03	<0.0001	1.348	0.50
		Error			35.209				1.113				1.325	
	Males	Line	3.09	<0.0001	28.355	0.51	2.74	<0.0001	0.919	0.46	3.17	<0.0001	1.353	0.52
		Error			27.121				1.057				1.248	
Starvation resistance	Sexes pooled	Sex	0.22	0.638	N/A		35.03	<0.0001	N/A		196.06	<0.0001	N/A	
		Line	1.86	<0.0001	5.472	0.29	1.55	0.001	0.500	0.08	2.39	<0.0001	2.522	0.37
		Sex×Line	2.12	<0.0001	6.756		1.03	0.392	0.048		2.3	<0.0001	2.055	
		Error			29.977				6.228				7.872	
	Females	Line	2.75	<0.0001	10.921	0.26	1.49	0.0001	0.828	0.10	3.79	<0.0001	5.913	0.36
		Error			31.217				7.26				10.570	
	Males	Line	3.34	<0.0001	13.454	0.32	1.35	0.005	0.499	0.09	4.11	<0.0001	3.211	0.38
		Error			28.736				4.791				5.170	

**b**			$\ln(CV_E)$				$\ln(MAD)$				$\ln(\sigma_E)$			
**Trait**	**Analysis**	**Source**	**F**	***P*-value**	σ^2	H^2	**F**	***P*-value**	σ^2	H^2	**F**	***P*-value**	σ^2	H^2
Chill coma recovery	Sexes pooled	Sex	56.48	<0.0001	N/A	0.63	13.43	0.0003	N/A	0.71	23.80	< 0.0001	N/A	0.75
		Line	6.22	<0.0001	0.091		7.07	<0.0001	0.209		9.81	< 0.0001	0.258	
		Sex×Line	1.22	0.064	0.006		1.45	0.002	0.021		1.33	0.013	0.015	
		Error			0.058				0.007				0.088	
	Females	Line	4.46	<0.0001	0.097	0.63	7.33	<0.0001	0.259	0.76	7.70	< 0.0001	0.281	0.77
		Error			0.056				0.082				0.084	
	Males	Line	4.27	<0.0001	0.098	0.62	4.72	<0.0001	0.201	0.65	6.79	< 0.0001	0.263	0.74
		Error			0.06				0.012				0.091	
Startle response	Sexes pooled	Sex	11.46	0.0009	N/A	0.51	12.13	0.0006	N/A	0.39	7.24	0.0077	N/A	0.54
		Line	10.45	<0.0001	0.122		4.67	<0.0001	0.079		6.94	< 0.0001	0.064	
		Sex×Line	0.54	1	-0.000		0.81	0.955	-0.000		0.78	0.973	-0.000	
		Error			0.095				0.107				0.055	
	Females	Line	2.90	<0.0001	0.102	0.49	2.00	<0.0001	0.06	0.33	3.08	< 0.0001	0.059	0.51
		Error			0.108				0.12				0.057	
	Males	Line	3.37	<0.0001	0.098	0.54	2.66	<0.0001	0.078	0.45	3.17	< 0.0001	0.056	0.52
		Error			0.083				0.094				0.052	
Starvation resistance	Sexes pooled	Sex	0.41	0.521	N/A	0.30	30.47	<0.0001	N/A	0.08	183.13	< 0.0001	N/A	0.36
		Line	1.74	<0.0001	0.015		1.46	0.004	0.009		2.48	< 0.0001	0.029	
		Sex×Line	2.28	<0.0001	0.023		1.05	0.310	0.002		2.22	< 0.0001	0.021	
		Error			0.091				0.139				0.088	
	Females	Line	2.91	<0.0001	0.037	0.28	1.41	0.001	0.014	0.08	3.83	< 0.0001	0.054	0.36
		Error			0.096				0.149				0.095	
	Males	Line	3.36	<0.0001	0.040	0.32	1.33	0.007	0.012	0.09	3.84	< 0.0001	0.047	0.36
		Error			0.004				0.124				0.082	

Table S4| Quantitative genetic analysis of variance in micro-environmental plasticity for males and females, pooled across traits. df: degrees of freedom; MS: Mean squares; F: F-ratio test; σ^2 (s.e.): variance component (standard error); H^2: broad sense heritability; r_{GT}: Cross-trait genetic correlation. Variance components given as -0.000 were negative.

**Analysis**	**Source**	**df**	**MS**	**F**	***P*-value**	σ^2	r_{GT}
Females	Trait	2	94.67	254.68	< 0.0001	N/A	0.04
	Line	204	0.40	1.10	0.2252	0.005	
	Trait×Line	365	0.37	4.33	< 0.0001	0.108	
	Error	1,161	0.09			0.086	
Males	Trait	2	40.70	112.34	< 0.0001	N/A	0.00
	Line	204	0.34	0.97	0.5794	-0.000	
	Trait×Line	365	0.36	4.68	< 0.0001	0.108	
	Error	1,160	0.08			0.078	

Data file S1| Data file used for the analyses. (**a**) Replicate means for ANOVA. (**b**) Pooled means for GWA analyses.

Data file S2| GWA analyses for ln(σ_E) of three quantitative traits. All variants significant at a nominal *P*-value < 10^{-5} in any test (females, males, sex average and sex difference) are listed. Effects are one-half the mean difference in ln(σ_E) between the major and minor allele classes.
